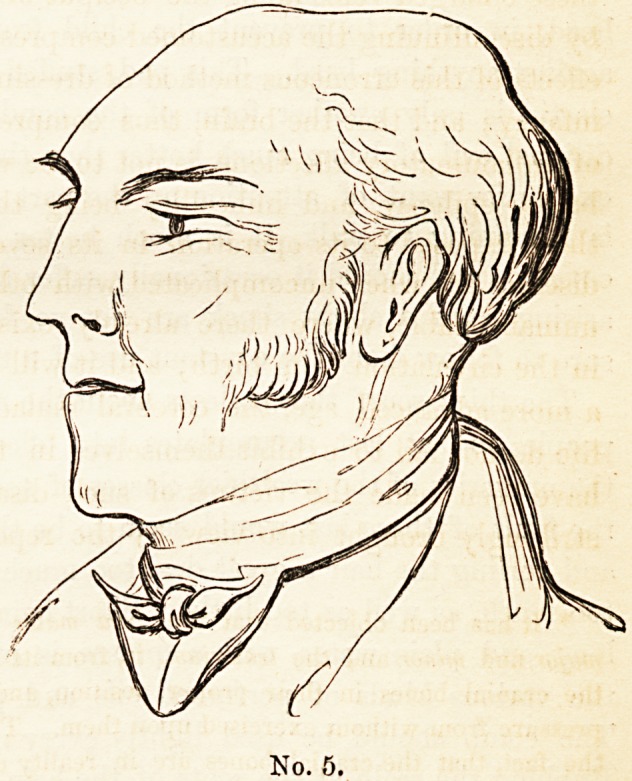# On Deformity of the Infantile Cranium

**Published:** 1849-01-01

**Authors:** 


ART. II.—*Influence des Vêtemens sur nos Organes. Déformation du Crâne, résultant de la méthode plus générale de Couvrir la Tête des Enfans.* Par le Docteur A. FOVILLE. Paris.

*The Influence of Clothing on our Organs. A Deformity of the Cranium, resulting from the common method of Covering the Heads of Infants.* By Dr. ACHILLE FOVILLE, Senior Physician to the Asile Départmental des Aliénés de la Seine Inférieure. Paris. pp. 69. With Illustrations.

FOVILLE, who had the charge of the *Asile Départmental de la Seine Inférieure,* paid great attention to the deformities of the cranium among the idiotic and imbecile patients confined within its walls; and he traced the origin of these cranial irregularities to mismanagement in infancy, especially that of bandaging the head too tightly. This deformity may be marked by drawing a line along the middle of the forehead, around over the ears, and beneath the protuberance of the occiput. This depression is most observable on the forehead and sides of the head. He accounts for it by tight bandaging in the early months of infancy. He observed it in all classes of society—among adults, old people, youths, children, and infants newly born—under such striking circumstances, that what was only a floating conjecture in his mind in 1829, was in 1834 a solid conviction. At a certain degree of intensity this malformation produces grave disturbances in the cerebral circulation, such as imbecility or epilepsy, and may, sooner or later, end in confirmed idiocy or insanity. It is a barbarity, he says, which would seem to have had its origin only among savages; and the object of his memoir, at the head of this article, is to lay open the magnitude of the evil, and to insist on the necessity of its being effectually remedied.

The shape of the head, when perfectly developed, is that of a sphere or spheroid, half of which is above the eyebrows and half below, the ear-hole being two-thirds of the distance in a line from the orbitar ridge to the occiput. The annexed outlines are examples of this kind of head, and, with such a formation, its fortunate possessor could not be anything else than intelligent, if not good. Every head, however, is not so well turned as these two are, but each, when left to nature, approximates to this type. The greater number of craniums are ovoid, and regularly rounded; and the variations or departures from this prevailing figure establish the difference between different individuals or races. A very little observation will show, that the best shaped heads may be slightly irregular, and frequently not quite symmetrical in their two halves. But the deformity described by Foville is a caricature of nature in her worst mode of expression, as will be seen in the annexed drawing, which is copied from one of Foville's illustrations.

The forehead retreats and is depressed, the sinciput bulges out into a knobby globe, the occiput is thrown back, and deeply indented just above the spinal column. In profile the outline is angular, which is contrary, not only to the line of beauty, but likewise to the sign of a healthy constitution, which is invariably curved and flowing. A practised eye will discern this singular deformity beneath a thick cap, or beneath a cap or bonnet, or detect it under the disguise of a thick crop of hair; but sometimes, in order to observe it, the head must be uncovered or shaved. There is, of course, every possible variety, but a very common inferiority of shape most people are familiar with in the example given in No. 5, which is a likeness. In the worst cases, the skull is divided into two portions, an upper and lower, by the circular depression, and the child in the night-cap (the subjoined outline) may serve to convey some idea of the miseries, both immediate and future, produced by drawing the bobbins rather too tightly. It will be remarked, that when this malformation is excessive, the forehead is thrown forward and the chin depressed, for the sake of preserving, it would seem, the balance of the median line.

Now, do such deformities arise from the process of ossification being disturbed? Is it rachitis of the cranium? or diseased softening of the bones? Evidently not; because these misshapen heads are met with in persons in all other respects healthy. In fact, the cranial bones are in these cases elongated *de seipsis*, proving an innate power of growth; whereas scrofulous bones are deficient in the power of growing. But is this deformity the result of external compression? It would seem to be so; although some authors affirm that no external force can alter the shape of the cranium. They judge by analogy, and ground their opinion on comparative anatomy. But, in this instance, comparative anatomy fails in deciding the question, which is one of fact, relating to the human head, that sometimes goes on enlarging till the fortieth year, and not to the lower animals, whose ossification is complete and stationary after a fœtation of a few weeks or months. Yet, though the analogy with the lower animals is unsatisfactory, we find that the comparison with other races of mankind is valid and conclusive. The Caribbees exhibit a remarkable proof in our favour, for their heads are from their birth forced into a false shape by a very mischievous artifice, the frontal bone being pressed almost flat, and the occiput squeezed out so much backwards, that one of these crania looks at first sight like the skull of a dog. Mr. Lawrence, in his work on the "Natural History of Man," has commented on this striking physiognomy; and Blumenbach, in his "Collectio Craniorum," gives two representations of it, which are worthy of attention. Exactly opposed to this kind of shape is that of the Peruvian skull, which, instead of being pressed out horizontally, is forced up vertically, into the shape of an obtuse cone. Foville says that Blumenbach mentions some Turkish skulls, exhibiting a circular depression, in consequence of ligatures having been tied round the head in infancy. M. Virey, in his article *Enfant* in the "Dictionnaire des Sciences Médicales," says it is certain that the shape of the head may be altered mechanically, and that some caps drawn tightly by ribbons force the head into a sugar-loaf shape; thus, he adds, producing idiotcy by means of compression.

In France, the rustics, if not the citizens, generally bandage their children's heads from the birth, exactly along the line of depression already pointed out; and it is remarkable, that the tip of the cartilage of the ear is, at that point where it is pressed upon, flattened and wasted, but that the lower portion of the ear, which has escaped the pressure, retains its original character. The scalp, likewise, over the fontanelle, is blanched, dry, and shining, exhibiting a few cicatrices, through which some scanty hairs make their appearance. Among adults, women suffer more than men, while children of either sex suffer equally; but then the women cover their heads more continually than the men, and the infants are all bandaged alike. These sad effects are entirely prevented by laying aside the head-dress from the first.

Some have supposed that the midwife may knead the head into a particular form during the act of birth. This is not the case: for the head of the child is always compressed and disfigured in a very awkward manner during the easiest labours, while, in tedious ones, it is distorted to a great degree. Nor does it recover its natural form immediately after birth, as it always does when the labour has been short and easy. It is not possible for us to compress the child's head at this time.

Those mothers who have been persuaded to discontinue the use of bandages acknowledge the happy results in consequence of their having done so; and others have remarked the ill effects of the bandage, although it has never occurred to them to abandon the use of it. No intelligent man, to whom Foville disclosed his views, ever entertained any doubts as to their reasonableness. Several medical men from Rouen, besides Dr. Hodgkin from London, MM. les Docteurs Marc and Pasquier, and Professor De Blainville, who visited his Asylum, agreed in his conclusions. It was the opinion of Pasquier, that the coincidence of the wasting of the gristle of the ear, and the atrophy of the hairy scalp, upon those parts over which the bandage or roller had evidently passed, was a proof positive in his favour. How, indeed, can these facts be denied, when the head is as deeply crimped by the marks of former bandaging, as the leg, above the knee, becomes permanently indented by the constant use of a tight garter?

The results obtained in studying the deformed heads at the *Asile Départmental de la Seine Infèrieure* bear out this view of the question. In the month of August, 1833, the number of patients there was 431, of whom 202 were men, and 229 women. Out of the total number of men, 109 heads were regular, and 93 deformed; of these 93, all did not betray the evidences of tight bandaging equally, for 36 were moderately marked—46 more distinctly, and 11 only very distinctly so. Out of the total number of women, there were 75 regular, and 154 deformed; and of the latter number, 68 moderately, 46 much more so, and 40 most of all. Relative differences apart, the sum total gives, out of 202 men, 109 regular conformations, and 93 deformed; while out of 229 women, it gives 75 regular conformations, and 154 deformed: both sexes taken together, it gives, out of the gross amount of 431 alienated, 184 regular conformations, and 247 deformities—*i. e.*, more than half. Among the men, the deformity does not extend to half the number, while among the women, the proportions exceed two-thirds—thus: Of both sexes taken together, it is 57 out of 100; of the men, 46 out of 100, and of the women 67 out of 100. From the 11th July, 1825, when the Asylum was first opened, up to the month of August, 1833, an interval of eight years, 508 men and 640 women were admitted, giving about one-sixth more of women than men; and the total differences between the two sexes are still the same—namely, about one-half of the men having deformed heads, and two-thirds of the women. This result is as interesting as it was unlooked for. But what is still more interesting than this is, that, in this Asylum, there are separate apartments for those variously affected—with fury, or moping, or passive mania—among the inmates. And besides this house in particular, there is a sort of town residence (*Maison Bourgeoise*), of a similar nature, reserved for ladies of fortune (*destinée aux dames pensionnaires de la première classe*), provided with three courts for the women, and five proper dormitories. In one of the courts are collected the incurables, who are the most indocile and violent; in two of the dormitories are enclosed the most brutal, and those the most incapable of occupying themselves; and in another dormitory are brought together, in company, the most laborious and sociable, as well as such as are the most disposed to the employment in common of sewing,* &c., &c.

Now it so happens, that this last dormitory presents the smallest proportion of deformed heads—14 out of 28, or half; whereas, in the two other dormitories, containing the most violent and indocile, and the most brutalized of the population, there are, out of 78 occupants, 58 badly-formed heads, or three-fourths. Let it be remarked, that the most brutal characters here present the worst-shaped heads—a result so much the more interesting, as the classification of disease has hitherto proceeded entirely regardless of the shape of the skull.

These observations, made by Foville at the Asylum, lead to the inquiry, whether their accuracy has been tested or corroborated in other establishments of the same kind, or whether one meets with such deformities in the world at large. He says, that when he was the *élève interne* of M. Esquirol, at the Salpêtrière, he had already remarked this malformation among the alienated in the wards of that hospital, and was sure of having, at that time, noticed the pernicious impress of the bandage around the cranium. It was the same at Charenton and Bicêtre; but then both these establishments frequently receive patients from the adjacent parts of Normandy. Dr. Delaye, a friend of Foville's, in charge of the *Hôpital des Aliénés* at Toulouse, confirms the notion, that the same evil is rife in the south of France as much as in the north—arising, in all probability, from the same cause. Many persons here, says Dr. Delaye, have their heads peaked (*pointue*), not only among the maniacal, but among the sane also. Children wear two caps, or cauls, bound round with a linen roller. These two cauls, or skull-caps, tied on with long ribbons, compress the head strongly, by being wound five or six times round very firmly; so that it is not uncommon

* The following note of Foville's is too good to be translated:—Qu'il me soit permis de faire connaître ici un resultat qu'aucune autre maison d'aliénés n'est peut-être pas parvenue, jusqu'à présent, à obtenir au même degré.

Les jardins spacieux de l'asile sont cultivés par les bras de nos hommes, dirigés par un jardinier et des infirmiers. Les travaux de la buanderie, ceux de la lingerie sont executés par les femmes aliénés, sous la direction de nos dames religieuses. Enfin, le transport des objets necessaires au service des bains de la cuisine est encore l'œuvre de nos insensés, et cela de quatre ans. Ainsi, nous avons pu utiliser, dans leur intérêts, ces malheureux auxquel le travail de corps est si favorable.—Op. Cit., p. 41.

to see persons with a depression or gutter along the circumference of their heads, exactly corresponding to the line of pressure. This indentation is deeply traced in some idiots and imbeciles in the *Hôpital des Aliénés de Toulouse.* It might be conjectured that these deplorable effects from the mode of bandaging infants' heads would be met with only among the poorer set of people; but this is not the case, for out of 40 persons of fortune (*pensionnaires pour les trois pensions supérieures*), 20 were thus deformed. The proportion is the same in each section of society; nor is this surprising, when we call to mind how many mothers put out their infants to wet-nurse, and, consequently, pass them over to the hands of the lowest and poorest of the population. Moreover, intelligent mothers do not feel themselves called upon to invent a new method of dressing their little ones, but take things as they find them, and do the best they can with what comes to hand.

Now, what are the injurious effects on the functions of the brain? This is a capital question, and the word that answers to the question will indicate the proper corrective of the evil.

The enormous proportion of badly formed heads in the asylum under Foville's care will suffice to show how closely such deformities are connected with mental derangement; and the relative differences between the two sexes, which gives so serious a preponderance against the women, adds to the importance of this deformity, when considered as an immediate or indirect cause of madness. It is an extensive question, which comprises not only the mad, but even those sensible folks who, with badly formed heads, go about their business apparently in the perfect possession of their faculties. The most simple disorders, however—such as headache and giddiness—may be all that arise from the use of the bandage, in some cases; although, in others, of a more serious character, they may manifest themselves as the warnings that precede and accompany the most dangerous forms of compression of the brain. Profound debility and a very deficient understanding are met with in such persons just as often as a slightly eccentric and an habitually irritable disposition—symptoms indicative of a troubled circulation through the encephalon. It is easily demonstrated: tie a string round the finger, and the blood is strangled at the tip; bind a roller round the compressible head of a child, and the course of the blood is impeded within the skull; for all the vessels of the head communicate freely with each other—the outside with the inside veins—the internal with the external carotids—the circulation anastamoses, conjoins, corresponds, and sympathises throughout every portion of the neck, face, skull, and brain. Only take into the account the unclosed opening of the fontanelle, beneath which flows the superior longitudinal sinus, and calculate how much this capacious channel must be engorged by pressure on the scalp;* for the external veins, first exposed to the pressure, empty themselves of their proper load, and force the burden back upon the sinuses of the brain, if not, further on, upon the sinuses or venous plexus of the spinal cord besides. The worst consequences are to be reckoned on.

One of the first effects of this sort of pressure externally is suppurative irritation of the hairy scalp. This portion of the skin, so highly vascular, is not merely bound down, but kept much too hot by means of bandages, thick caps, or bonnets. The perspiration is both increased and obstructed, the hair falls off, and, in the dirtier people, it is quickly infested with insects.† A sero-purulent discharge escapes, which is popularly regarded as beneficial; and so it is, by relieving the internal congestion. The cervical glands enlarge, and a train of scrofulous symptoms ensue, owing entirely to mismanagement from the first. Foville says, that he has seen the compression cause a varicose condition of the external veins of the head, and he gives a drawing of one of these enlarged veins along the occiput of an infant, which was relieved by discontinuing the accustomed compress. Such are the most evident effects of this erroneous method of dressing the head, especially during infancy; and that the brain, thus compressed, should become the seat of inflammatory affections, is not to be wondered at—meningitis, cerebritis, epilepsy, and imbecility being the most frequent maladies in those exposed to its operation in its severest form. Terrible as these diseases are when uncomplicated with other evils, they become almost unmanageable, where there already exists some permanent difficulty in the circulation from birth; and it will be easily understood, that, at a more advanced age, the cerebral maladies peculiar to this epoch of life do not fail to exhibit themselves in those unfortunate beings who have been made the victims of such disastrous nursing,—a fact most strikingly brought into view by the reports of the *Asile des Aliénés,*

* It has been objected that the *dura mater* with its processes called the *falx major* and *minor* and the *tentorium*, is, from its inelastic nature, sufficient to retain the cranial bones in their proper position, and to counteract the effects of any pressure from without exercised upon them. The only answer to this objection is the fact, that the cranial bones are in reality distorted and pushed awry, in the manner so ably pointed out by Foville in the above-cited work.

† The loathsome insects mentioned by Foville are seldom met with in this country, except among the lowest of the low. "It is impossible (says one of the City missionaries) to convey a just idea of their state; the quantities of vermin are amazing. I have entered a room, and in a few minutes I have felt them dropping on my hat from the ceiling like peas. 'They may be gathered by handfuls,' observed one of the inmates."—*Parliamentary Reports: Lord Ashley's Speech on Emigration and Ragged Schools, June* 5, 1848.

quoted above. It is only by studying these statistical reports that we are enabled to appreciate this inquiry at its full value.

The cause of the evil being thus detected and proved, it only remains to discover and apply the remedy. Nothing can be easier. Tell the rudest mother in the world that her mode of nursing is doing mischief to her children, and show her how and in what manner she is inflicting a permanent injury upon them, and you have already won over her tenderest affections to your side, and gone more than half way in effecting the radical reform so earnestly solicited. The ordinary night-cap, in this country, is tied exactly round that part of the head which Foville has pointed out as the seat of pressure, and if it do not produce such extravagant deformities as those of which he has given us several drawings, one of which we annex, it at least helps in preventing the proper development of the cranium, and may become a means of rendering many a head less happily shapen than it would have been of its own accord, had it been left to the care of Dame Nature alone. How can the tender, pulsating head of an infant, through which half the blood of the whole body is flowing, sustain with impunity the tightness of a common worked lace cap, nicely tied on in the most approved nursery fashion? Is it not evident, that the head of a newly-born child ought to be handled with the utmost delicacy, and that every sort of pressure ought to be most carefully removed from it? It requires time and space to evolve, grow, dilate, and expand into the round cranium of a capacious understanding.

No. 5.

But, besides these mischievous caps and execrable head-rollers, there are other articles of dress, not less pernicious than they are, in daily use; such as thick bonnets made to "*fit well*," tied under the chin, covering and pressing on the ears, and heating the head,—pieces of oiled-silk, for the purpose of preventing the perspiration transuding and soiling the silk or straw above, stitched inside, and worn precisely over the great fontanelle. Nothing can be worse: for the head-dress of a child ought to be light, simple, and just sufficient to preserve it from the weather, and it ought to be airy at the same time. Children, if left to themselves, run about without hats or bonnets, like the poorer urchins, who have never a bonnet or hat to wear, except a thick pole of tangled locks, which is nature's own covering. It will scarcely be credited, by *lay* readers, that a common straw or silk bonnet made too hot, or too tight, just as the fashion may direct, will tend to alter the shape of the head, prevent its growth, damage the intellect, and lay the foundation for eventual disease of the brain. The form of the head is never so pleasing as when it has been allowed to grow up without interference. In general, all classes keep the head too much covered. It is one cause of baldness in adults, and of difficult dentition in children. A light silk net or thin bonnet is sufficient in the first months after birth; and some months later, it is better to let the head go uncovered, except with something light to protect it from the rain or sunshine during the day, or from the chilliness of night during sleep.

There is a fashion, almost out of date, of putting a pad about the head, in order to prevent the child from stunning itself by falling against anything hard. This pad, stuffed with wool, quilted, and made elastic, is only another form of the condemned bandage, with the additional evil of being much hotter than the flannel roller. It is a mere excuse for want of attention on the part of the nurse. When a child is beginning to walk, it is much better to let the head remain uncovered; and should it occasionally suffer from a fall, the inconveniences arising from a slight shock are not so formidable as those which are sure to follow from the constant use of a thick heating bandage.

The thick scurf that collects about the roots of the hair in the poorer people, and at last accumulates into offensive scabs, is seldom seen in the nurseries of the wealthier classes of society in this country—indeed, the fault of these last would seem to be that of washing, and combing, and curling the hair a great deal too much; for the hair may be dressed too much, as well as too little. Short hair, in early life, is preferable to long, and plain water, with a sponge, is more beneficial than soap and the various kinds of perfumed oils and pomatums so much in vogue. The comb should be used lightly every morning, and then the brush. Rubbing the scalp at the roots of the hair, brushing too forcibly, greasy applications, hot curling irons, and tight curling papers, &c., which only serve to irritate the scalp, ought never to be employed. A very soft brush, gently applied, does good in an infant before the hair is grown, but when it is once grown up, the hair-brush is an indispensable article of the dressing-table for the rest of life. Cutting the points of the hair frequently is a good practice, except that it renders it coarse, but strong at the same time. The ends of the cut hair are exhalent surfaces, which keep the head cool. The dress of very young children should be both light and warm, easily secured without pins, which prick and tease the skin, and the child should be soused in tepid water daily, and rubbed dry quickly. Such are Foville's invaluable remarks on the management of children, but especially on the evil effects of a heavy head-dress.

## Figures and Tables

**Figure f1:**
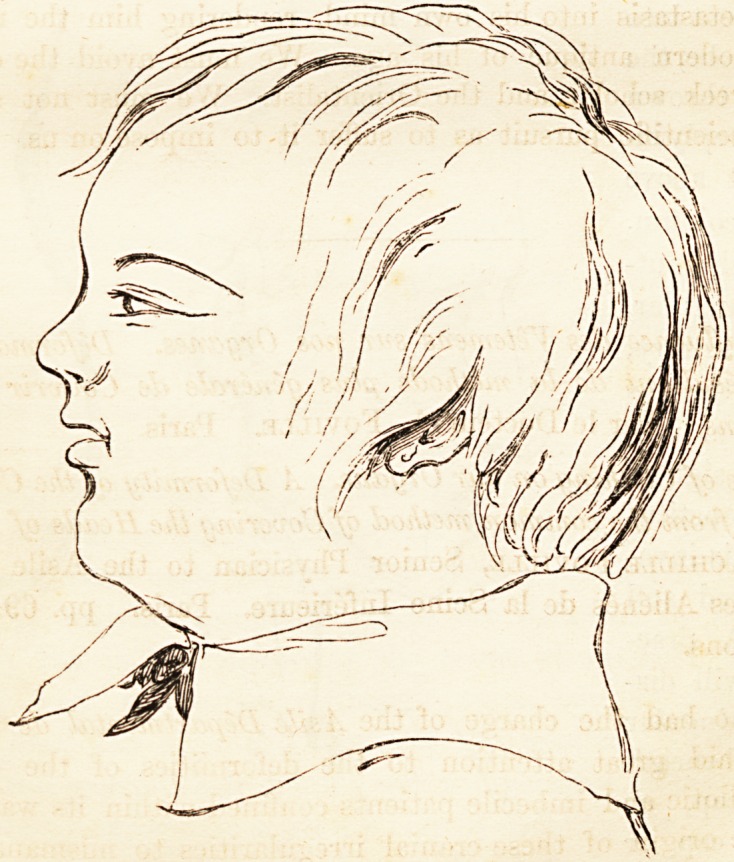


**Figure f2:**
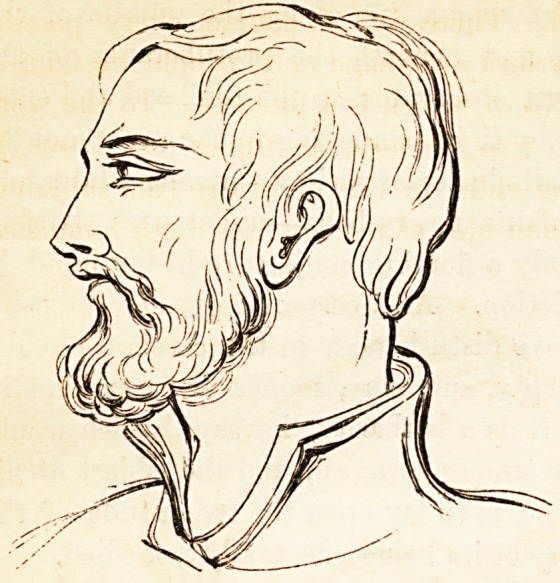


**Figure f3:**
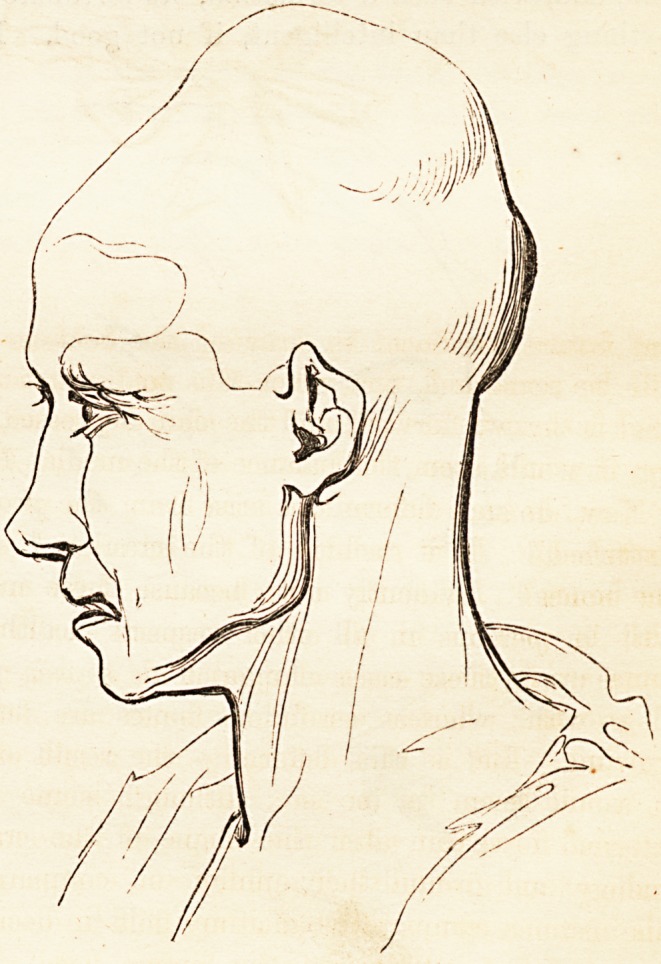


**Figure f4:**
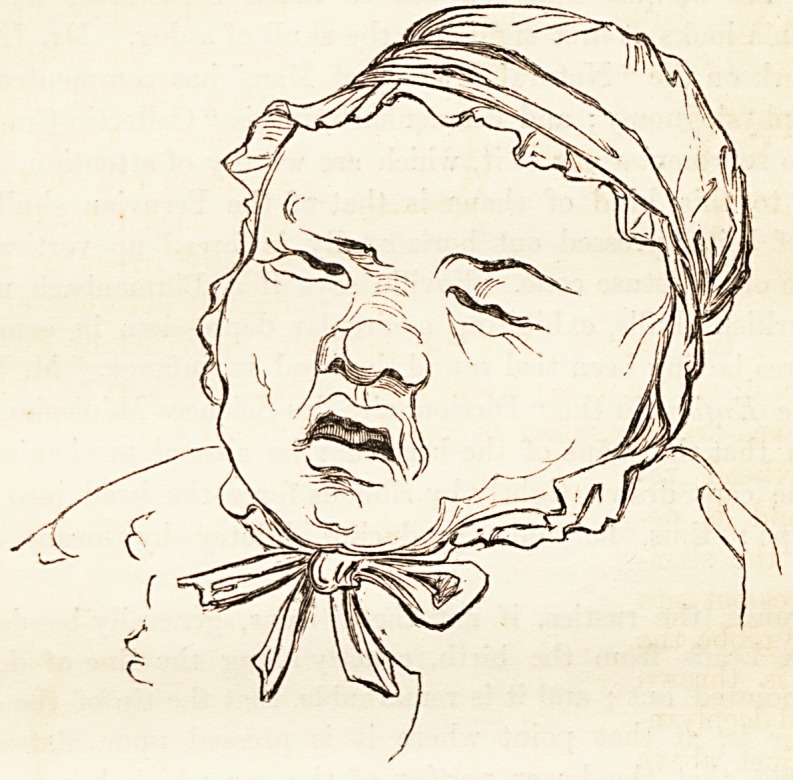


**No. 5. f5:**